# Comparison of Machine Learning Algorithms for Predicting Hospital Readmissions and Worsening Heart Failure Events in Patients With Heart Failure With Reduced Ejection Fraction: Modeling Study

**DOI:** 10.2196/41775

**Published:** 2023-04-17

**Authors:** Boshu Ru, Xi Tan, Yu Liu, Kartik Kannapur, Dheepan Ramanan, Garin Kessler, Dominik Lautsch, Gregg Fonarow

**Affiliations:** 1 Merck & Co, Inc Rahway, NJ United States; 2 Amazon Web Services Inc Seattle, WA United States; 3 School of Continuing Studies Georgetown University Washington, DC United States; 4 Ahmanson-UCLA Cardiomyopathy Center University of California, Los Angeles Los Angeles, CA United States

**Keywords:** deep learning, machine learning, hospital readmission, heart failure, heart failure with reduced ejection fraction, worsening heart failure event, Bidirectional Encoder Representations From Transformers, BERT, clinical registry, medical claims, real-world data

## Abstract

**Background:**

Heart failure (HF) is highly prevalent in the United States. Approximately one-third to one-half of HF cases are categorized as HF with reduced ejection fraction (HFrEF). Patients with HFrEF are at risk of worsening HF, have a high risk of adverse outcomes, and experience higher health care use and costs. Therefore, it is crucial to identify patients with HFrEF who are at high risk of subsequent events after HF hospitalization.

**Objective:**

Machine learning (ML) has been used to predict HF-related outcomes. The objective of this study was to compare different ML prediction models and feature construction methods to predict 30-, 90-, and 365-day hospital readmissions and worsening HF events (WHFEs).

**Methods:**

We used the Veradigm PINNACLE outpatient registry linked to Symphony Health’s Integrated Dataverse data from July 1, 2013, to September 30, 2017. Adults with a confirmed diagnosis of HFrEF and HF-related hospitalization were included. WHFEs were defined as HF-related hospitalizations or outpatient intravenous diuretic use within 1 year of the first HF hospitalization. We used different approaches to construct ML features from clinical codes, including frequencies of clinical classification software (CCS) categories, Bidirectional Encoder Representations From Transformers (BERT) trained with CCS sequences (BERT + CCS), BERT trained on raw clinical codes (BERT + raw), and prespecified features based on clinical knowledge. A multilayer perceptron neural network, extreme gradient boosting (XGBoost), random forest, and logistic regression prediction models were applied and compared.

**Results:**

A total of 30,687 adult patients with HFrEF were included in the analysis; 11.41% (3184/27,917) of adults experienced a hospital readmission within 30 days of their first HF hospitalization, and nearly half (9231/21,562, 42.81%) of the patients experienced at least 1 WHFE within 1 year after HF hospitalization. The prediction models and feature combinations with the best area under the receiver operating characteristic curve (AUC) for each outcome were XGBoost with CCS frequency (AUC=0.595) for 30-day readmission, random forest with CCS frequency (AUC=0.630) for 90-day readmission, XGBoost with CCS frequency (AUC=0.649) for 365-day readmission, and XGBoost with CCS frequency (AUC=0.640) for WHFEs. Our ML models could discriminate between readmission and WHFE among patients with HFrEF. Our model performance was mediocre, especially for the 30-day readmission events, most likely owing to limitations of the data, including an imbalance between positive and negative cases and high missing rates of many clinical variables and outcome definitions.

**Conclusions:**

We predicted readmissions and WHFEs after HF hospitalizations in patients with HFrEF. Features identified by data-driven approaches may be comparable with those identified by clinical domain knowledge. Future work may be warranted to validate and improve the models using more longitudinal electronic health records that are complete, are comprehensive, and have a longer follow-up time.

## Introduction

Heart failure (HF), defined by the US Centers for Disease Control and Prevention as a condition when the heart cannot pump enough blood and oxygen to support other organs in one’s body [1], is highly prevalent in the United States, affecting approximately 6 million Americans aged ≥20 years [2]. HF represents a major and growing public health concern in the United States. Between 2008 and 2018, hospitalizations owing to HF increased by 20% from 1,060,540 to 1,270,360 [3]. A systematic review of medical costs associated with HF in the United States found that the annual median total medical costs for HF were estimated at US $24,383 per patient between 2014 and 2020 [4] with total annual costs of US $43.6 billion in 2020 [5].

Approximately 31% to 56% of HF cases in the United States are classified as HF with reduced ejection fraction (HFrEF) [6-8], defined as a left ventricular ejection fraction of ≤40% [9]. Patients with HFrEF represent a subset of patients with HF with substantial morbidity and mortality. Patients with HFrEF are also at risk of worsening HF events (WHFEs, including outpatient intravenous [IV] diuretic use or HF-related hospitalization) [10,11]. Patients with a WHFE have a high risk of adverse outcomes and substantially higher health care use and costs than those without a WHFE [10,11].

The 30-day readmission rate has been used as an important quality of care measure to evaluate hospital performance, and through the Hospital Readmissions Reduction Program, the Centers for Medicare & Medicaid Services have penalized hospitals with higher 30-day readmission rates of >US $3 billion [12]. A 2021 study using HF hospitalizations from 2010 to 2017 in the National Readmission database found that among patients with HFrEF who had an HF hospitalization, approximately 18.1% had a 30-day all-cause readmission [13]. A 2011 to 2014 database analysis of patients with HFrEF found that 56% of patients with HFrEF with WHFE were readmitted within 30 days of the WHFE [11].

It is crucial for providers and payers to identify patients with HF who are at high risk of readmission and WHFEs and to provide targeted interventions in an attempt to prevent these adverse events from occurring. However, predictive model performance for readmission after HF hospitalization remains unsatisfactory, and it is substantially worse than that of models that predict mortality [14].

Machine learning (ML) [15] has been applied to predict HF-related outcomes, and most ML models (76%) have outperformed conventional statistical models [16]. One major advantage of ML models is that they do not require statistical assumptions that are usually too strict for real-world data. Because semantic relationships between medical codes can be complicated (eg, is-a, synonym, equivalent, or overlapping), they can void statistical assumptions regarding independence. Furthermore, the granularity of medical codes often causes extraordinarily high dimensionality of the search space, making models more vulnerable to overfitting. Deep learning (DL), a state-of-the-art ML method [15], has the additional advantage of not requiring labor-intensive feature engineering and data preprocessing. Owing to these advantages, ML methods, including DL, have become popular in health outcome prediction research [17-19].

Most current ML prediction models [16,20-23] are limited by (1) being developed using single-center data and lacking external validation or (2) focusing on general HF or other disease indications in which the disease progression trajectory is clinically different from HFrEF. Furthermore, limited evidence is available on how DL works in the area of HF as a feature extraction method and how traditional and neural network models perform using different types of features. This study aims to compare different ML models to predict 30-day, 90-day, and 365-day hospital readmissions and WHFEs after HF hospitalization among patients with HFrEF using a nationally representative US-based HF registry linked to claims data.

## Methods

### Study Design and Data Sources

The study was conducted by analyzing a US database linking the Veradigm PINNACLE outpatient registry with Symphony Health’s Integrated Dataverse (IDV) pharmacy and medical claims data from July 1, 2013, to September 30, 2017. The PINNACLE registry is cardiology’s largest outpatient quality improvement registry, which captures data on coronary artery disease, hypertension, HF, and atrial fibrillation. PINNACLE contains information on patient demographics, diagnoses and comorbidities, cardiovascular events, vital signs, HF symptoms, laboratory orders and results, medications, and death date [24]. The Symphony IDV data set includes physician office medical claims, hospital claims, and pharmacy claims. These claims were preadjudicated and submitted by providers to different types of payers in the United States.

**Figure 1:**
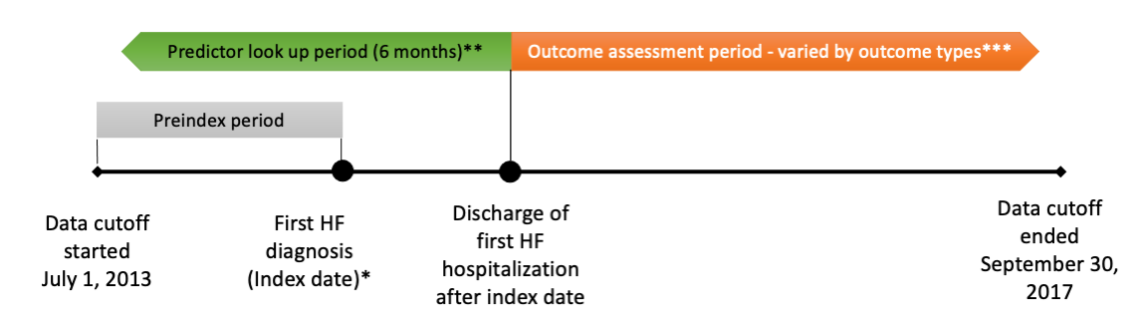
Study design. HF: heart failure. *Index date can be any date between January 1, 2014, and September 30, 2017. **The predictor look-up period ended at the discharge date of the first HF hospitalization after the index date and started at 6 months before the end date. The start of the predictor look-up period could be prior to, on, or after the index date. ***The outcome assessment period began at the end of the predictor look-up period. The length varied (30, 90, and 365 days) by outcome type.

The date of the first documentation of HF diagnosis was set as the index date for each patient (Figure 1). The time interval before the index date within the study period was considered the preindex period. The admission date of the first HF hospitalization was either on or after the index date. The period after the discharge date of the first HF hospitalization was considered the outcome assessment period. The period before the discharge date of the first HF hospitalization was defined as the predictor lookup period. We chose 6 months as the length of the predictor lookup period. While 6 and 12 months are both common selections in retrospective outcome research, we chose the shorter period to increase the available number of patients for model training and evaluation. Only the predictor variables observed during the predictor lookup period were used in the training and evaluation of the prediction models.

### Ethical Considerations

As this study was a retrospective study on existing deidentified data, it was exempt from institutional review board review as determined by the WIRB-Copernicus Group, Inc (WCG) Institutional Review Board (Work order # 1-1435573-1).

### Study Population

Patients were included if they met the following criteria: (1) had a diagnosis of HF in the registry, with HFrEF confirmed by the presence of either an ejection fraction <45% or at least 2 claims of the HFrEF diagnosis using the International Classification of Diseases, Tenth Revision (ICD-10) codes I50.2X or I50.4X or ICD-9 code 428.2X; (2) had their first diagnosis of HF (index date) between January 1, 2014, and September 30, 2016; (3) were aged ≥18 years on the index date; (4) had at least 1 medical claim and 1 pharmacy claim in the preindex period and at least 1 medical claim and 1 pharmacy claim after the index date; and (5) had an HF-related hospitalization on or after the index date.

Patients with the following diagnoses or procedures in the preindex period were excluded: clinical trial participation, heart transplant, left ventricular assist device, adult congenital heart disease (eg, single-ventricle disease), and amyloidosis. Multimedia Appendix 1 presents a flowchart to obtain the final sample.

For WHFEs, the discharge date of the first HF hospitalization was between the index date and September 30, 2016 (to ensure the availability of 1 year of follow-up time within the study period). For 30-day readmission, the discharge date of the first HF hospitalization was between the index date and August 31, 2017. For 90-day readmission, the discharge date of the first HF hospitalization was between the index date and July 2, 2017. For 365-day readmission, the discharge date of the first HF hospitalization was between the index date and September 30, 2016.

### Outcome Measures

The outcomes included 30-day, 90-day, and 365-day hospital readmission as well as WHFEs. Recent clinical studies [25-27] on HF, specifically the HFrEF subtype, have been performed in a population of patients with worsening HF who are at increased risk for subsequent all-cause and HF-related hospitalization and death.

WHFEs were defined as HF-related hospitalizations or outpatient IV diuretic use in the year after the first HF hospitalization. Any hospital claims with a primary diagnosis of HF using ICD-10 codes I50.1, I50.2x, I50.3x, I50.4x, I50.8x, I50.9, or I11.0; ICD-9 codes 402.01, 402.11, 402.91, or 428.XX; or a record of hospital admission with a primary reason for HF in the registry was considered an HF-related hospitalization. IV diuretic use was identified using either the registry records or procedure codes in claims (J1205, J1940, J3265, S0171, or S9361). Sensitivity analyses were conducted to study the composite outcome of a WHFE or death.

An individual would be categorized as “yes” or “positive” for 30-day readmission if the first hospital claim of a subsequent hospitalization was within 30 days of the discharge date of the previous HF-related hospitalization. We excluded patients who neither belonged to the positive class nor had any records on or beyond 30 days after the previously mentioned discharge date. Then, we categorized the response of remaining patients as “no” or “negative.” Similar definitions were applied to 90- and 365-day readmissions, using the 90- and 365-day periods to measure readmission. For the analyses of 30-day and 90-day readmission, we excluded those who died within 30 days or 90 days after the discharge date. For the analysis of 365-day readmission, we conducted sensitivity analyses to study the composite outcome of readmission or death. Additional sensitivity analyses were conducted to exclude planned readmissions.

### Predictors and Feature Engineering

Demographics and health care use included sociodemographic factors (age as a continuous variable, gender, race, ethnicity, and health insurance) and health care use in the preindex period (the number of all-cause hospitalizations, number of all-cause emergency room visits, and number of all-cause outpatient visits).

Medical diagnoses and procedures and drugs were identified using the ICD-9 and ICD-10 codes, procedure codes, and national drug codes (NDCs).

Clinical attributes included (as available in the Veradigm PINNACLE outpatient registry): alcohol use, tobacco use, HF education completed or documented, HF plan of care (yes/no), New York Heart Association functional classification for HF, left ventricular ejection fraction, HF symptoms, physical assessment, quality of life measures, height, weight, BMI, heart rate, sodium, potassium, B-type natriuretic peptide, N-terminal pro B–type natriuretic peptide, Hemoglobin A_1c_, low-density lipoprotein cholesterol, high-density lipoprotein cholesterol, triglycerides, total cholesterol, systolic blood pressure, diastolic blood pressure, serum creatinine, creatinine clearance, estimated glomerular filtration rate, international normalized ratio, amylase levels, alanine transaminase, aspartate transaminase, direct bilirubin, total bilirubin, cystatin-C, high-sensitivity C-reactive protein, thyroid-stimulating hormone, hemoglobin, hematocrit, platelet count, and white blood cell count.

### Feature Engineering for Medical Diagnoses, Procedures, and Drugs

We investigated 4 approaches for building ML features from medical diagnoses, procedures, and drug records.

1. Clinical classification software (CCS) frequency: first, we reduced the dimensionality of the predictors and aggregated the medical codes at different granularities. We converted diagnosis and procedure codes into the Agency for Healthcare Research and Quality’s CCS categories [28] and mapped NDC codes onto the top-level anatomical components of the body, as defined by the World Health Organization Anatomic Therapeutic Chemical (ATC) classification system [29]. Approaches similar to ours have been used in recent studies. For example, Chen et al [30] aggregated diagnosis and procedure codes into CCS categories and generic drug names into ATC therapeutic subclasses [31,32]. Denny et al [33] transformed the ICD diagnosis codes to PheCodes for phenotype groups specified in the PheWAS project [30-32]. Specifically, we made modifications to the NDC-ATC mapping table based on the study by Kury et al [34] by assigning drugs with multiple therapeutic target areas to a new category that combines all target areas (eg, if drug A treats both blood forming organs [ATC category B] and the cardiovascular system [ATC category C], it was categorized as “BC”). We then built a frequency table with CCS and ATC categories as columns, where the values in the rows represent the number of times a CCS- or ATC-relevant event had happened to the patient in the predictor lookup period. This is a traditional feature engineering approach often used in data mining competitions and industrial projects and has been criticized for not preserving sequential patterns and causing high sparsity.
2. Bidirectional Encoder Representations from Transformers (BERT) + CCS: BERT is a deep neural network model that achieves state-of-the-art performance in multiple natural language processing (NLP) tasks [35]. Considering that both human written texts (eg, novels and news) and patient clinical records are recorded in the form of sequential events, recent research has explored the use of BERT to represent patient medical records [36,37]. In this study, we adopted the BERT model and the Hugging-Face Transformer Python package [38] to process the sequences of medical code records into vectors during the predictor look-up period. The BERT+CCS models were first pretrained on claims of 298,284 patients with HF in the Merative MarketScan 2011 to 2020 Commercial and Medicare Databases, with medical codes converted to the CCS and ATC categories found in approach 1. The models were then fine-tuned on sequences of the CCS and ATC categories from the PINNACLE+IDV data. To avoid information leakage, data for patients used in the validation and testing were not used to fine-tune the BERT model. We configured the hidden size of BERT to 64, hidden layers to 4, attention heads to 4, and intermediate size to 256 to control the total number of model parameters. We used the output of the pooling layer of the fine-tuned BERT model to map patient sequences of medical codes to a fixed-length feature vector, which was 64 dimensions per sequence and 256 dimensions per patient (for a total of 4 sequences, each covering 1.5 months of the predictor look-up period).
3. BERT + raw: Similarly, we pretrained and fine-tuned another type of BERT model using the sequence of medical codes obtained from the original (also known as the raw format of) ICD-9/ICD-10 codes, procedure codes, and ATC categories (derived from NDC codes).
4. Prespecified features: we built features from groups of diagnoses, procedures, and drug codes recognized by clinical science experts as risk factors of the WHFE based on clinical knowledge and previous literature [39].

### Feature Engineering for Clinical Attributes

We converted numerical clinical attribute measurements available in the Veradigm PINNACLE outpatient registry into a frequency table, in which the values in the rows represented the number of times a laboratory test was taken in the predictor look-up period. We did not use the measurement results directly for the following reasons: (1) missing information on normal range, assay type, collection route, and many other details prevented the accurate normalization of results from different laboratory facilities; (2) high sparsity in many variables; and (3) orders of nonroutine laboratory tests suggest that physicians were suspicious of certain disease conditions, and simply using the frequency can preserve such insight. We used the latest value in each predictor look-up period for nominal clinical attribute measures.

### Prediction Models

We used a multilayer perceptron neural network model [40] with 3 fully connected layers (256, 128, and 64 neurons), 2 dropout and batch normalization layers, and a sigmoid function for prediction as well as extreme gradient boosting (XGBoost) [41], random forest [42], and logistic regression [43] models. For each type, we trained 4 models corresponding to the 4 feature engineering approaches mentioned above.

Patients with each outcome were randomly divided into the training (70%), validation (15%), and testing (15%) data sets. We used the training and validation data to maximize the area under the receiver operating characteristic curve (AUC) to select the optimal value for the following hyperparameters: learning rate and dropout rate for multilayer perceptron neural network; number of trees, eta, max depth, colsample by tree, and minimum child weight for XGBoost; number of trees, maximum features, and maximum depth for random forest; and C (penalty strength) for logistic regression. To address the class imbalance issue in the data, we adopt a cost-sensitive learning approach by setting the weight of the negative class to the prevalence of the positive class in the training data set. We also investigated assigning different class weights and synthetic oversampling methods using the adaptive synthetic sampling approach for imbalanced learning (ADASYN) [44], but these methods were not superior to our original approach.

We evaluated the prediction models and feature engineering approaches in terms of AUC and area under the precision-recall curve (AUPR) using the testing data set. Although AUC was more commonly reported in recent research, we listed both because some studies argued that AUPR was more informative on imbalanced data sets [45]. We also included the precision and recall scores of each model after converting the predicted probability >0.5 to a positive prediction. Precision and recall were derived from the proportions of true positive (TP), false positive, true negative, and false negative predictions, by definitions of precision as *TP / (TP + false positive)* and recall as *TP / (TP – false negative)*.

## Results

A total of 30,687 adults with HFrEF were included in the analysis (Table 1; Multimedia Appendix 1). The average age was 70.1 years, and most patients (18,206/30,687, 59.32%) were male (Table 1). The rates of 30-, 90-, and 365-day readmission rates were 11.41% (3186/27,917), 21.09% (5792/27,455), and 38.93% (8394/21,562), respectively (Multimedia Appendix 2). In all, 42.81% (9231/21,562) of patients experienced at least one WHFE within 1 year after HF hospitalization (Multimedia Appendix 2). Note that 99.22% (2770/27,917) of patients were excluded from the 30-day readmission calculations because of a lack of follow-up on or beyond 30 days (see the *Methods* section, *Outcome Measures*). For the same reason, 11.77% (3232/27,455) of patients were excluded from the 90-day readmission outcome analyses, and 42.31% (9125/21,562) of patients were excluded from the 365-day readmission and WHFE analyses.

**Table 1:** Descriptive results of patient characteristics and outcome measures (N=30,687)^a^.

		All patients (N=30,687)	30-day readmission^b^			WHFE^c^	
			Yes (n=3186)	No (n=24,731)	Yes (n=9231)		No (n=12,331)
**Age (years)**							
	Value, mean (SD)	70.1 (13.0)	69.7 (13.2)	70.1 (13.0)	69.4 (13.3)		70.4 (12.9)
	Value, median (IQR)	72 (62-80)	71 (61-80)	72 (62-80)	71 (61-80)		72 (62-80)
**Gender, n (%)**							
	Female	12,481 (40.67)	1337 (41.96)	10,082 (40.77)	3927 (42.54)		4895 (39.69)
	Male	18,206 (59.33)	1849 (58.03)	14,649 (59.23)	5304 (57.46)		7436 (60.30)
**Race, n (%)**							
	Asian	241 (0.78)	20 (0.62)	209 (0.84)	70 (0.75)		110 (0.89)
	Black or African American	3705 (12.07)	401 (12.59)	3010 (12.17)	1307 (14.16)		1340 (10.86)
	Other	143 (0.47)	11 (0.34)	125 (0.51)	37 (0.40)		73 (0.6)
	Unknown	9389 (30.59)	988 (31.01)	7590 (30.69)	2866 (31.05)		3811 (30.91)
	White	17,209 (56.08)	1766 (55.43)	13,797 (55.79)	4951 (53.63)		6997 (56.74)
**Ethnicity, n (%)**							
	Hispanic	1275 (4.15)	159 (4.9)	1009 (4.08)	427 (4.62)		478 (3.87)
	Non-Hispanic	677 (2.21)	77 (2.42)	564 (2.28)	230 (2.49)		333 (2.70)
	Unknown	28,735 (93.64)	2950 (92.59)	23,158 (93.64)	8574 (92.88)		11,520 (93.42)
**Health insurance, n (%)**							
	Medicaid	786 (2.56)	89 (2.8)	645 (2.61)	281 (3.04)		285 (2.3)
	Medicare	4894 (15.95)	474 (14.87)	3944 (15.95)	1423 (15.42)		1919 (15.56)
	Private	4666 (15.21)	482 (15.13)	3826 (15.47)	1330 (14.41)		1964 (15.92)
	Other	133 (0.43)	11 (0.34)	107 (0.43)	38 (0.4)		52 (0.4)
	Multiple^d^	9243 (30.12)	981 (30.79)	7325 (29.62)	2757 (29.86)		3605 (29.23)
	No insurance	156 (0.51)	18 (0.56)	125 (0.51)	55 (0.6)		55 (0.4)
	Unknown	10,809 (35.22)	1131 (35.49)	8759 (35.42)	3347 (36.25)		3811 (30.91)

^a^Some percentages may not add up to 100%, owing to rounding. Percentages were calculated using the number of available respondents as the denominator.

^b^90.27% (2770/30,687) of patients were not included in the yes and no categories of 30-day readmission outcomes owing to no follow-up on or beyond 30 days after the first hospitalization for heart failure.

^c^WHFE: worsening heart failure event.

^d^If a patient had health insurance plans in at least 2 categories among Medicaid, Medicare, Private, and Other, the patient was classified in the “multiple” category.

The prediction model and feature engineering approach combinations with the best AUC for each outcome were XGBoost with CCS frequency (0.595) for 30-day readmission, random forest with CCS frequency (0.630) for 90-day readmission, XGBoost with CCS frequency (0.649) for 365-day readmission, and XGBoost with CCS frequency (0.640) for WHFE (Table 2).

**Table 2:** Primary analysis results of machine learning prediction models to predict 30-day, 90-day, and 365-day hospital readmission, as well as worsening heart failure events (WHFEs) after heart failure hospitalizations.

Prediction model and medical code processing method				AUC^a^		AUPR^b^		Precision		Recall
**30-day hospital readmission**										
	**Logistic regression**									
		CCS^c^ frequency	*0.580* ^d^		*0.129* ^d^		*0.154* ^d^		0.381	
		BERT^e^ + CCS	0.491		0.114		0.114		0.448	
		BERT + raw^f^	0.561		0.123		0.133		*0.490* ^d^	
		Prespecified	0.568		0.126		0.141		0.436	
	**MLP NN^g^**									
		CCS frequency	*0.583* ^d^		0.131		0.157		0.385	
		BERT + CCS	0.548		0.121		0.130		0.425	
		BERT + raw	0.554		0.122		0.135		*0.445* ^d^	
		Prespecified	0.565		*0.133* ^h^		*0.161* ^h^		0.407	
	**Random forest**									
		CCS frequency	*0.577* ^d^		0.125		0.142		*0.400* ^d^	
		BERT + CCS	0.576		0.125		0.141		0.391	
		BERT + raw	0.565		*0.127* ^d^		*0.148* ^d^		0.377	
		Prespecified	0.566		0.126		0.146		0.371	
	**XGBoost^i^**									
		CCS frequency	*0.595* ^h^		0.130		*0.145* ^d^		0.513	
		BERT + CCS	0.592		*0.131* ^d^		*0.145* ^d^		*0.525* ^h^	
		BERT + raw	0.570		0.128		*0.145* ^d^		0.454	
		Prespecified	0.570		0.123		0.135		0.432	
**90-day hospital readmission**										
	**Logistic regression**									
		CCS frequency	*0.616* ^d^		0.255		*0.307* ^d^		0.457	
		BERT + CCS	0.500		0.216^d^		0.220		*0.568* ^h^	
		BERT + raw	0.606		*0.243* ^d^		0.272		0.534	
		Prespecified	0.588		0.238		0.263		0.524	
	**MLP NN**									
		CCS frequency	0.593		*0.244* ^d^		*0.301* ^d^		0.365	
		BERT + CCS	0.573		0.229		0.261		0.364	
		BERT + raw	*0.607* ^d^		0.243		0.269		*0.544* ^d^	
		Prespecified	0.590		0.240		0.277		0.433	
	**Random forest**									
		CCS frequency	*0.630* ^h^		*0.263* ^h^		*0.324* ^h^		0.457	
		BERT + CCS	0.594		0.235		0.255		*0.537* ^d^	
		BERT + raw	0.572		0.225		0.276		0.209	
		Prespecified	0.611		0.248		0.289		0.475	
	**XGBoost**									
		CCS frequency	*0.625* ^d^		*0.252* ^d^		*0.294* ^d^		*0.498* ^d^	
		BERT + CCS	0.587		0.232		0.253		0.493	
		BERT + raw	0.571		0.233		0.272		0.364	
		Prespecified	0.605		0.243		0.277		0.487	
**365-day hospital readmission**										
	**Logistic regression**									
		CCS frequency	*0.611* ^d^		*0.439* ^h^		*0.500* ^d^		0.445	
		BERT + CCS	0.557		0.418		0.444		0.519	
		BERT + raw	0.607		0.434		0.471		*0.542* ^d^	
		Prespecified	0.591		0.421		0.457		0.475	
	**MLP NN**									
		CCS frequency	*0.616* ^d^		0.380		0.434		0.488	
		BERT + CCS	0.590		0.426		0.470		0.457	
		BERT + raw	0.605		*0.429* ^d^		*0.503* ^d^		0.352	
		Prespecified	0.589		0.414		0.419		*0.813* ^d^	
	**Random forest**									
		CCS frequency	*0.617* ^d^		*0.436* ^d^		0.519		0.357	
		BERT + CCS	0.588		0.416		0.507		0.225	
		BERT + raw	0.597		0.430		0.474		*0.481* ^d^	
		Prespecified	0.606		0.430		*0.572* ^h^		0.224	
	**XGBoost**									
		CCS frequency	*0.649* ^h^		0.395		0.448		*0.561* ^d^	
		BERT + CCS	0.615		*0.435* ^d^		*0.480* ^d^		0.504	
		BERT + raw	0.600		0.432		0.473		0.514	
		Prespecified	0.608		0.432		0.475		0.498	
**WHFE**										
	**Logistic regression**									
		CCS frequency	*0.625* ^d^		*0.484* ^d^		*0.549* ^d^		0.462	
		BERT + CCS	0.509		0.439		0.444		*0.661* ^h^	
		BERT + raw	0.606		0.472		0.506		0.560	
		Prespecified	0.595		0.472		0.517		0.490	
	**MLP NN**									
		CCS frequency	*0.606* ^d^		*0.478* ^d^		*0.540* ^d^		0.449	
		BERT + CCS	0.605		0.476		0.521		0.512	
		BERT + raw	0.596		0.460		0.479		*0.625* ^d^	
		Prespecified	0.596		0.470		0.520		0.459	
	**Random forest**									
		CCS frequency	*0.633* ^d^		*0.486* ^d^		0.565		0.419	
		BERT + CCS	0.619		0.471		*0.592* ^h^		0.264	
		BERT + raw	0.573		0.455		0.508		0.338	
		Prespecified	0.608		0.475		0.514		*0.542* ^d^	
	**XGBoost**									
		CCS frequency	*0.640* ^h^		*0.489* ^h^		*0.542* ^d^		0.536	
		BERT + CCS	0.615		0.473		0.508		*0.563* ^d^	
		BERT + raw	0.596		0.465		0.497		0.538	
		Prespecified	0.609		0.473		0.514		0.520	

^a^AUC: area under the receiver operating characteristic curve.

^b^AUPR: area under the precision-recall curve.

^c^CCS: clinical classification software.

^d^The highest score among the 4 medical code processing methods for the specific model and outcome.

^e^BERT: Bidirectional Encoder Representations From Transformers.

^f^raw: original clinical codes in the data source.

^g^MLP NN: multilayer perceptron neural network.

^h^The highest score among all models and medical code processing methods for a specific outcome.

^i^XGBoost: extreme gradient boosting.

Except when predicting 90-day readmission, the XGBoost prediction models generally performed better than the other models. Within a given prediction model, the tree-based ensemble and boosting algorithms and logistic regression all achieved a higher AUC with CCS frequency features than other medical code processing methods. Features extracted by data-driven medical code processing approaches (CCS frequency, BERT + CCS, and BERT + raw) may be comparable to features prespecified by clinical domain knowledge.

Similar findings were observed in the sensitivity analyses of unplanned readmissions or outcomes including death (Table 3).

**Table 3:** Sensitivity analysis results of machine learning prediction models patients with heart failure with reduced ejection fraction.

Prediction model and medical code processing method				AUC^a^		AUPR^b^		Precision		Recall
**30-day unplanned readmission**										
	**Logistic regression**									
		CCS^c^ frequency	*0.579* ^d^		0.125		0.149		0.376	
		BERT^e^ + CCS	0.490		0.110		0.109		0.441	
		BERT + raw^f^	0.555		0.120		0.130		*0.490* ^d^	
		Prespecified	0.568		*0.129* ^d^		*0.150* ^d^		0.468	
	**MLP NN^g^**									
		CCS frequency	0.564		0.124		0.137		0.497	
		BERT + CCS	0.549		0.118		0.125		0.508	
		BERT + raw	0.554		0.119		0.124		*0.596* ^h^	
		Prespecified	*0.574* ^d^		*0.130* ^h^		*0.158* ^h^		0.403	
	**Random forest**									
		CCS frequency	*0.576* ^d^		0.121		0.137		*0.396* ^d^	
		BERT + CCS	0.558		0.118		0.130		0.376	
		BERT + raw	0.571		*0.124* ^d^		*0.145* ^d^		0.383	
		Prespecified	0.563		*0.124* ^d^		*0.145* ^d^		0.388	
	**XGBoost^i^**									
		CCS frequency	*0.591* ^h^		*0.126* ^d^		*0.140* ^d^		*0.510* ^d^	
		BERT + CCS	0.575		*0.126* ^d^		*0.140* ^d^		0.501	
		BERT + raw	0.563		0.123		0.138		0.452	
		Prespecified	0.568		0.122		0.136		0.455	
**90-day unplanned readmission**										
	**Logistic regression**									
		CCS frequency	*0.615* ^d^		0.251		*0.300* ^d^		0.462	
		BERT + CCS	0.498		0.213		0.216		*0.562* ^d^	
		BERT + raw	0.603		*0.237* ^d^		0.264		0.523	
		Prespecified	0.592		0.232		0.264		0.432	
	**MLP NN**									
		CCS frequency	0.600		*0.242* ^d^		0.276		*0.498* ^h^	
		BERT + CCS	0.579		0.237		*0.277* ^d^		0.413	
		BERT + raw	*0.603* ^d^		0.239		0.275		0.466	
		Prespecified	0.586		0.231		0.267		0.379	
	**Random forest**									
		CCS frequency	*0.629* ^h^		*0.256* ^d^		0.316		0.444	
		BERT + CCS	0.577		0.209		*0.333* ^h^		0.001	
		BERT + raw	0.597		0.231		0.314		0.213	
		Prespecified	0.611		0.246		0.288		*0.472* ^d^	
	**XGBoost**									
		CCS frequency	*0.620* ^d^		*0.252* ^h^		*0.295* ^d^		0.502	
		BERT + CCS	0.588		0.233		0.257		*0.510* ^d^	
		BERT + raw	0.605		0.240		0.279		0.440	
		Prespecified	0.605		0.245		0.281		0.499	
**365-day unplanned readmission**										
	**Logistic regression**									
		CCS frequency	*0.614* ^d^		*0.436* ^d^		*0.498* ^d^		0.450	
		BERT + CCS	0.560		0.415		0.442		0.531	
		BERT + raw	0.606		0.428		0.465		*0.541* ^h^	
		Prespecified	0.593		0.420		0.461		0.466	
	**MLP NN**									
		CCS frequency	0.589		0.419		0.480		0.363	
		BERT + CCS	0.589		0.419		0.458		*0.472* ^d^	
		BERT + raw	*0.601* ^d^		0.421		0.536		0.238	
		Prespecified	0.587		*0.425* ^d^		*0.498* ^d^		0.351	
	**Random forest**									
		CCS frequency	*0.614* ^d^		*0.427* ^d^		*0.612* ^h^		0.185	
		BERT + CCS	0.587		0.411		0.606		0.117	
		BERT + raw	0.595		0.409		0.535		0.159	
		Prespecified	0.608		0.425		0.474		*0.450* ^d^	
	**XGBoost**									
		CCS frequency	*0.620* ^h^		*0.436* ^h^		*0.482* ^d^		*0.530* ^d^	
		BERT + CCS	0.599		0.419		0.450		0.529	
		BERT + raw	0.607		0.426		0.463		0.525	
		Prespecified	0.607		0.428		0.472		0.494	
**365-day readmission or death**										
	**Logistic regression**									
		CCS frequency	*0.611* ^d^		*0.469* ^d^		*0.526* ^d^		0.447	
		BERT + CCS	0.549		0.445		0.477		0.398	
		BERT + raw	0.607		0.467		0.503		*0.551* ^d^	
		Prespecified	0.586		0.454		0.487		0.493	
	**MLP NN**									
		CCS frequency	0.590		*0.465* ^d^		*0.513* ^d^		0.462	
		BERT + CCS	0.594		0.458		0.504		0.435	
		BERT + raw	*0.607* ^d^		0.462		0.484		0.647	
		Prespecified	0.589		0.452		0.467		*0.651* ^h^	
	**Random forest**									
		CCS frequency	*0.613* ^d^		*0.468* ^d^		0.570		0.303	
		BERT + CCS	0.593		0.450		0.556		0.206	
		BERT + raw	0.603		0.454		*0.576* ^h^		0.203	
		Prespecified	0.607		0.462		0.507		*0.467* ^d^	
	**XGBoost**									
		CCS frequency	*0.623* ^h^		*0.474* ^h^		*0.520* ^d^		0.530	
		BERT + CCS	0.599		0.464		0.499		*0.540* ^d^	
		BERT + raw	0.599		0.459		0.495		0.507	
		Prespecified	0.606		0.465		0.506		0.504	
**WHFE^j^ or death**										
	**Logistic regression**									
		CCS frequency	*0.625* ^d^		*0.484* ^d^		*0.549* ^d^		0.462	
		BERT + CCS	0.509		0.439		0.444		*0.661* ^h^	
		BERT + raw	0.606		0.472		0.506		0.560	
		Prespecified	0.595		0.472		0.517		0.490	
	**MLP NN**									
		CCS frequency	*0.606* ^d^		*0.478* ^d^		*0.540* ^d^		0.449	
		BERT + CCS	0.605		0.476		0.521		0.512	
		BERT + raw	0.596		0.460		0.479		*0.625* ^d^	
		Prespecified	0.596		0.470		0.520		0.459	
	**Random forest**									
		CCS frequency	*0.633* ^d^		*0.486* ^d^		0.565		0.419	
		BERT + CCS	0.619		0.471		*0.592* ^h^		0.264	
		BERT + raw	0.573		0.455		0.508		0.338	
		Prespecified	0.608		0.475		0.514		*0.542* ^d^	
	**XGBoost**									
		CCS frequency	*0.640* ^h^		*0.489* ^h^		*0.542* ^d^		0.536	
		BERT + CCS	0.615		0.473		0.508		*0.563* ^d^	
		BERT + raw	0.596		0.465		0.497		0.538	
		Prespecified	0.609		0.473		0.514		0.520	

^a^AUC: area under the receiver operating characteristic curve.

^b^AUPR: area under the precision-recall curve.

^c^CCS: clinical classification software.

^d^The highest score among the 4 medical code processing methods for the specific model and outcome.

^e^BERT: Bidirectional Encoder Representations From Transformers.

^f^raw: the original clinical codes from data source were used.

^g^MLP NN: multilayer perceptron neural network.

^h^The highest score among all models and medical code processing methods for a specific outcome.

^i^XGBoost: Extreme Gradient Boosting.

^j^WHFE: worsening heart failure events.

## Discussion

### Principal Findings

This study used one of the most commonly used outpatient registries for HF, the Veradigm PINNACLE outpatient registry linked to the Symphony IDV medical claims data set, to predict readmissions and WHFEs after HF hospitalizations in patients with HFrEF. This study is among the first to use DL/ML approaches to help identify a population at high risk for HFrEF by predicting an array of subsequent adverse events among patients with HFrEF after HF hospitalization. Most importantly, this study provided a comprehensive overview and comparison of different feature engineering approaches for predicting these HF outcomes, including their respective combinations: BERT, CCS, and the use of raw codes (raw). In particular, it was innovative to experiment with different combinations of feature engineering approaches and ML prediction models. We found that ML features constructed by data-driven approaches, including CCS frequency, BERT+CCS, and BERT+raw, performed on par with and, for some prediction algorithms and outcomes, better than feature engineering plans specified by clinical domain knowledge. Tree-based ensemble and boosting prediction algorithms with raw diagnosis and procedure codes converted to frequencies of CCS categories achieved higher AUCs than other combinations of algorithms and features for all tasks.

BERT is among the most contemporary NLP models for embedding medical codes and representing patient temporal clinical records in a matrix form for downstream analyses [36,37]. Interest in its application in the medical field is surging [17,46,47]. To feed this data-hungry model for this particular study, we reduced the layers and dimensions of BERT and pretrained the model on a large administrative claims data set of the Merative MarketScan 2011 to 2020 Commercial and Medicare Databases. We found that the prediction models using BERT features were not superior to those using CCS frequency features. This may be attributed to the differences between medical codes and natural languages. BERT often outperforms term-frequency methods in NLP tasks, where the sequence and context of tokens carry important signals [35]. However, it may not have been advantageous in this study because the order and context of standardized code data (eg, diagnosis, procedure, and drug code records) collected over a relatively short predictor look-up period may not be relevant to readmission and WHFE risks.

Despite the use of various prediction algorithms and feature engineering combinations, the model performance in this study was moderate across the primary analysis outcomes. The AUC and AUPR differences in our various algorithms and feature engineering combinations may be too close to draw deterministic conclusions. In our previous study using administrative claims data [39], we found that a bidirectional long short-term memory model with medical embedding features from the NLP model Word2Vec outperformed traditional ML models with prespecified features in predicting 30 days (AUC 0.597 vs 0.510) and 90-day readmission (AUC 0.614 vs 0.509) in patients with HFrEF. Although in this study we attempted to use an HF registry database with more detailed clinical information to further improve the prediction model performance, the results did not meet this expectation. Besides the data type and sources, this study also differed from the previous study in terms of the length of the predictor look-up period and the chosen prediction models. The PINNACLE+IDV data provide more detailed clinical information for patients with HFrEF; however, there are some limitations regarding the data source that may have prevented the models from achieving higher performance. The PINNACLE registry data were voluntarily reported by participating physicians in the outpatient setting, which may not capture all current and historical clinical information and health care events. In particular, the high missingness of laboratory variables during the predictor look-up window prevented our models from fully using these clinically known risk factors. IDV data may not capture all payers and all encounters for each patient either. For example, the readmission rates shown in this study seem to be lower than previously reported national averages [13], which may reflect a failure to capture all readmissions and potentially lower-risk patients or better care for those followed up at PINNACLE sites.

### Challenges and Future Work

Similar to previous studies [18,39,48,49], we still found it challenging to predict 30-day readmission following HF hospitalization in patients with HFrEF, even using more advanced DL models and a database with detailed clinical information. Apart from the data limitations discussed above, there are several plausible reasons for this. The first is the imbalance between positive and negative cases, which causes ML models to be insufficiently trained to learn generalizable patterns related to the outcome of interest. We attempted to resolve this by training the BERT models without using the outcomes and using cost-sensitive learning by configuring the class weight parameters of the models. We also failed to further improve the model performance using ADASYN [44]. Another issue is the definition of 30-day readmission, which currently categorizes any patient readmitted on or after the 31st day after discharge into the negative class. The 30-day readmission measure may not be an ideal indicator of clinical risk for an individual patient, as it may also be linked to factors that are not well captured in this study, such as the social determinants of health, hospital administration, provider practice, and other unknown factors [50]. In addition, it seems that prediction models performed better using single-center data because this avoids the issue of interoperability challenges across different health care systems and can also facilitate the collection of more detailed patient-, provider-, and facility-level information [51]. Therefore, future research is warranted to validate and further improve the model using longitudinal electronic health records, which are more complete, comprehensive, and have longer follow-up times. From a modeling perspective, graph and network structure-based patient representation learning algorithms have been reported in recent research [52,53], which have the potential to surpass data insufficiency and injecting medical knowledge into them can be another direction for further investigation.

### Conclusions

These results demonstrate that ML models provide modest discrimination of HF events in patients with HFrEF. This study also suggests that features constructed by data-driven approaches may be comparable to those specified by clinical domain knowledge. Despite our modeling and feature construction efforts, predicting readmission and WHFEs after HF hospitalization remains a challenging task. Future work may benefit from using more complete and comprehensive data as well as adopting additional patient representation learning methods.

## Appendix

Multimedia Appendix 1 Patient flow diagram.

Multimedia Appendix 2 Descriptive results of outcome measures.

## Abbreviations

ADASYN: adaptive synthetic sampling approach for imbalanced learning
ATC: Anatomical Therapeutic Chemical
AUC: area under the receiver operating characteristic curve
AUPR: area under the precision-recall curve
BERT: bidirectional encoder representations from transformers
CCS: clinical classification software
DL: deep learning
HF: heart failure
HFrEF: heart failure with reduced ejection fraction
ICC-10: International Classification of Diseases, Tenth Revision
IDV: Integrated Dataverse
IV: intravenous
ML: machine learning
NDC: national drug code
NLP: natural language processing
TP: true positive
WCG: WIRB-Copernicus Group, Inc
WHFE: worsening heart failure event
XGBoost: extreme gradient boosting

## Data Availability

The Veradigm PINNACLE outpatient registry data, Symphony Health’s Integrated Dataverse (IDV) claims data, and the Merative MarketScan 2011 to 2020 Commercial and Medicare Databases that support the findings of this study were commercially licensed from corresponding data vendors and are not available.

## References

[1] (2022). Heart failure. Centers for Disease Control and Prevention.

[2] Tsao CW, Aday AW, Almarzooq ZI, Alonso A, Beaton AZ, Bittencourt MS, Boehme AK, Buxton AE, Carson AP, Commodore-Mensah Y, Elkind MS, Evenson KR, Eze-Nliam C, Ferguson JF, Generoso G, Ho JE, Kalani R, Khan SS, Kissela BM, Knutson KL, Levine DA, Lewis TT, Liu J, Loop MS, Ma J, Mussolino ME, Navaneethan SD, Perak AM, Poudel R, Rezk-Hanna M, Roth GA, Schroeder EB, Shah SH, Thacker EL, VanWagner LB, Virani SS, Voecks JH, Wang N, Yaffe K, Martin SS (2022). Heart disease and stroke statistics-2022 update: a report from the American Heart Association. Circulation.

[3] Clark KA, Reinhardt SW, Chouairi F, Miller PE, Kay B, Fuery M, Guha A, Ahmad T, Desai NR (2022). Trends in heart failure hospitalizations in the US from 2008 to 2018. J Card Fail.

[4] Urbich M, Globe G, Pantiri K, Heisen M, Bennison C, Wirtz HS, Di Tanna GL (2020). A systematic review of medical costs associated with heart failure in the USA (2014-2020). Pharmacoeconomics.

[5] Heidenreich PA, Albert NM, Allen LA, Bluemke DA, Butler J, Fonarow GC, Ikonomidis JS, Khavjou O, Konstam MA, Maddox TM, Nichol G, Pham M, Piña IL, Trogdon JG, American Heart Association Advocacy Coordinating Committee, Council on Arteriosclerosis‚ Thrombosis and Vascular Biology, Council on Cardiovascular Radiology and Intervention, Council on Clinical Cardiology, Council on Epidemiology and Prevention, Stroke Council (2013). Forecasting the impact of heart failure in the United States: a policy statement from the American Heart Association. Circ Heart Fail.

[6] Ibrahim NE, Song Y, Cannon CP, Doros G, Russo P, Ponirakis A, Alexanian C, Januzzi JL (2019). Heart failure with mid-range ejection fraction: characterization of patients from the PINNACLE Registry®. ESC Heart Fail.

[7] Tsao CW, Lyass A, Enserro D, Larson MG, Ho JE, Kizer JR, Gottdiener JS, Psaty BM, Vasan RS (2018). Temporal trends in the incidence of and mortality associated with heart failure with preserved and reduced ejection fraction. JACC Heart Fail.

[8] Vasan RS, Xanthakis V, Lyass A, Andersson C, Tsao C, Cheng S, Aragam J, Benjamin EJ, Larson MG (2018). Epidemiology of left ventricular systolic dysfunction and heart failure in the framingham study: an echocardiographic study over 3 decades. JACC Cardiovasc Imaging.

[9] Murphy SP, Ibrahim NE, Januzzi JL (2020). Heart failure with reduced ejection fraction: a review. JAMA.

[10] Butler J, Djatche LM, Sawhney B, Chakladar S, Yang L, Brady JE, Yang M (2020). Clinical and economic burden of chronic heart failure and reduced ejection fraction following a worsening heart failure event. Adv Ther.

[11] Butler J, Yang M, Manzi MA, Hess GP, Patel MJ, Rhodes T, Givertz MM (2019). Clinical course of patients with worsening heart failure with reduced ejection fraction. J Am Coll Cardiol.

[12] Wadhera RK, Joynt Maddox KE, Desai NR, Landon BE, Md MV, Gilstrap LG, Shen C, Yeh RW (2021). Evaluation of hospital performance using the excess days in acute care measure in the hospital readmissions reduction program. Ann Intern Med.

[13] Khan MS, Sreenivasan J, Lateef N, Abougergi MS, Greene SJ, Ahmad T, Anker SD, Fonarow GC, Butler J (2021). Trends in 30- and 90-day readmission rates for heart failure. Circ Heart Fail.

[14] Ouwerkerk W, Voors AA, Zwinderman AH (2014). Factors influencing the predictive power of models for predicting mortality and/or heart failure hospitalization in patients with heart failure. JACC Heart Fail.

[15] Goodfellow I, Bengio Y, Courville A, Goodfellow I, Bengio Y, Courville A (2016). Machine learning basics. Deep Learning.

[16] Shin S, Austin PC, Ross HJ, Abdel-Qadir H, Freitas C, Tomlinson G, Chicco D, Mahendiran M, Lawler PR, Billia F, Gramolini A, Epelman S, Wang B, Lee DS (2021). Machine learning vs. conventional statistical models for predicting heart failure readmission and mortality. ESC Heart Fail.

[17] Si Y, Du J, Li Z, Jiang X, Miller T, Wang F, Jim Zheng W, Roberts K (2021). Deep representation learning of patient data from Electronic Health Records (EHR): a systematic review. J Biomed Inform.

[18] Min X, Yu B, Wang F (2019). Predictive modeling of the hospital readmission risk from patients' claims data using machine learning: a case study on COPD. Sci Rep.

[19] Masum S, Hopgood A, Stefan S, Flashman K, Khan J (2022). Data analytics and artificial intelligence in predicting length of stay, readmission, and mortality: a population-based study of surgical management of colorectal cancer. Discov Oncol.

[20] Sarijaloo F, Park J, Zhong X, Wokhlu A (2021). Predicting 90 day acute heart failure readmission and death using machine learning-supported decision analysis. Clin Cardiol.

[21] Zhao P, Yoo I, Naqvi SH (2021). Early prediction of unplanned 30-day hospital readmission: model development and retrospective data analysis. JMIR Med Inform.

[22] Gopukumar D, Ghoshal A, Zhao H (2022). Predicting readmission charges billed by hospitals: machine learning approach. JMIR Med Inform.

[23] Lv H, Yang X, Wang B, Wang S, Du X, Tan Q, Hao Z, Liu Y, Yan J, Xia Y (2021). Machine learning-driven models to predict prognostic outcomes in patients hospitalized with heart failure using electronic health records: retrospective study. J Med Internet Res.

[24] Partner registries. The National Cardiovascular Data Registry (NCDR), American College of Cardiology.

[25] A study of vericiguat in participants with heart failure with reduced ejection fraction (HFrEF) (MK-1242-001) (VICTORIA). ClinicalTrials.gov.

[26] Registrational study with omecamtiv mecarbil (AMG 423) to treat chronic heart failure with reduced ejection fraction (GALACTIC-HF). ClinicalTrials.gov.

[27] Effect of sotagliflozin on cardiovascular events in participants with type 2 diabetes post worsening heart failure (SOLOIST-WHF Trial). ClinicalTrials.gov.

[28] Software tools. Agency for Healthcare Research and Quality.

[29] WHO Collaborating Centre for Drug Statistics Methodology Structure and principles. ATC.

[30] Chen R, Stewart WF, Sun J, Ng K, Yan X (2019). Recurrent neural networks for early detection of heart failure from longitudinal electronic health record data: implications for temporal modeling with respect to time before diagnosis, data density, data quantity, and data type. Circ Cardiovasc Qual Outcome.

[31] Wu P, Gifford A, Meng X, Li X, Campbell H, Varley T, Zhao J, Carroll R, Bastarache L, Denny J, Theodoratou E, Wei WQ (2019). Mapping ICD-10 and ICD-10-CM codes to phecodes: workflow development and initial evaluation. JMIR Med Inform.

[32] Wei W, Bastarache LA, Carroll RJ, Marlo JE, Osterman TJ, Gamazon ER, Cox NJ, Roden DM, Denny JC (2017). Evaluating phecodes, clinical classification software, and ICD-9-CM codes for phenome-wide association studies in the electronic health record. PLoS One.

[33] Denny JC, Ritchie MD, Basford MA, Pulley JM, Bastarache L, Brown-Gentry K, Wang D, Masys DR, Roden DM, Crawford DC (2010). PheWAS: demonstrating the feasibility of a phenome-wide scan to discover gene-disease associations. Bioinformatics.

[34] Kury F, Bodenreider O (2017). Mapping U.S. FDA national drug codes to anatomical-therapeutic-chemical classes using RxNorm. AMIA Annu Symp Proc.

[35] Devlin J, Chang M, Lee K, Toutanova K (2019). BERT: pre-training of deep bidirectional transformers for language understanding. Proceedings of the 2019 Conference of the North American Chapter of the Association for Computational Linguistics: Human Language Technologies.

[36] Li Y, Rao S, Solares JR, Hassaine A, Ramakrishnan R, Canoy D, Zhu Y, Rahimi K, Salimi-Khorshidi G (2020). BEHRT: transformer for electronic health records. Sci Rep.

[37] Rasmy L, Xiang Y, Xie Z, Tao C, Zhi D (2021). Med-BERT: pretrained contextualized embeddings on large-scale structured electronic health records for disease prediction. NPJ Digit Med.

[38] Wolf T, Chaumond J, Debut L, Sanh V, Delangue C, Moi A, Cistac P, Rault T, Louf R, Funtowicz M, Davison J, Shleifer S, von Platen P, Ma C, Jernite Y, Plu J, Xu C, Scao TL, Gugger S, Drame M, Lhoest Q, Rush A (2020). Transformers: state-of-the-art natural language processing. Proceedings of the 2020 Conference on Empirical Methods in Natural Language Processing: System Demonstrations.

[39] Wang Z, Chen X, Tan X, Yang L, Kannapur K, Vincent JL, Kessler GN, Ru B, Yang M (2021). Using deep learning to identify high-risk patients with heart failure with reduced ejection fraction. J Health Econ Outcomes Res.

[40] Hastie T, Tibshirani R, Friedman J (2009). The Elements of Statistical Learning Data Mining, Inference, and Prediction.

[41] Chen T, Guestrin C (2016). XGBoost: a scalable tree boosting system. Proceedings of the 22nd ACM SIGKDD International Conference on Knowledge Discovery and Data Mining.

[42] Breiman L (2001). Random forests. Mach Learn.

[43] Hosmer JD, Lemeshow S, Sturdivant R (2013). Applied Logistic Regression. 3rd edition.

[44] He H, Bai Y, Garcia E, Li S (2008). ADASYN: adaptive synthetic sampling approach for imbalanced learning. Proceedings of the IEEE International Joint Conference on Neural Networks (IEEE World Congress on Computational Intelligence).

[45] Saito T, Rehmsmeier M (2015). The precision-recall plot is more informative than the ROC plot when evaluating binary classifiers on imbalanced datasets. PLoS One.

[46] Ormerod M, Martínez Del Rincón J, Devereux B (2021). Predicting semantic similarity between clinical sentence pairs using transformer models: evaluation and representational analysis. JMIR Med Inform.

[47] Mohammadi R, Jain S, Namin AT, Scholem Heller M, Palacholla R, Kamarthi S, Wallace B (2020). Predicting unplanned readmissions following a hip or knee arthroplasty: retrospective observational study. JMIR Med Inform.

[48] Frizzell JD, Liang L, Schulte PJ, Yancy CW, Heidenreich PA, Hernandez AF, Bhatt DL, Fonarow GC, Laskey WK (2017). Prediction of 30-day all-cause readmissions in patients hospitalized for heart failure: comparison of machine learning and other statistical approaches. JAMA Cardiol.

[49] Awan SE, Bennamoun M, Sohel F, Sanfilippo FM, Dwivedi G (2019). Machine learning-based prediction of heart failure readmission or death: implications of choosing the right model and the right metrics. ESC Heart Fail.

[50] Olsen CR, Mentz RJ, Anstrom KJ, Page D, Patel PA (2020). Clinical applications of machine learning in the diagnosis, classification, and prediction of heart failure. Am Heart J.

[51] Mahajan SM, Heidenreich P, Abbott B, Newton A, Ward D (2018). Predictive models for identifying risk of readmission after index hospitalization for heart failure: a systematic review. Eur J Cardiovasc Nurs.

[52] Choi E, Bahadori MT, Song L, Stewart WF, Sun J (2017). GRAM: graph-based attention model for healthcare representation learning. KDD.

[53] Li MM, Huang K, Zitnik M (2022). Graph representation learning in biomedicine and healthcare. Nat Biomed Eng.

