# Supplementary material for: Comparison of Machine Learning Algorithms for Predicting Hospital Readmissions and Worsening Heart Failure Events in Patients With Heart Failure With Reduced Ejection Fraction: Modeling Study
Source: JMIR Form Res. 2023 Apr 17;7:e41775. doi: 10.2196/41775 (PMC10152335; doi:10.2196/41775)
Supplement: Multimedia Appendix 1 [file formative_v7i1e41775_app1.docx]

Appendix 1. Patient flow diagram


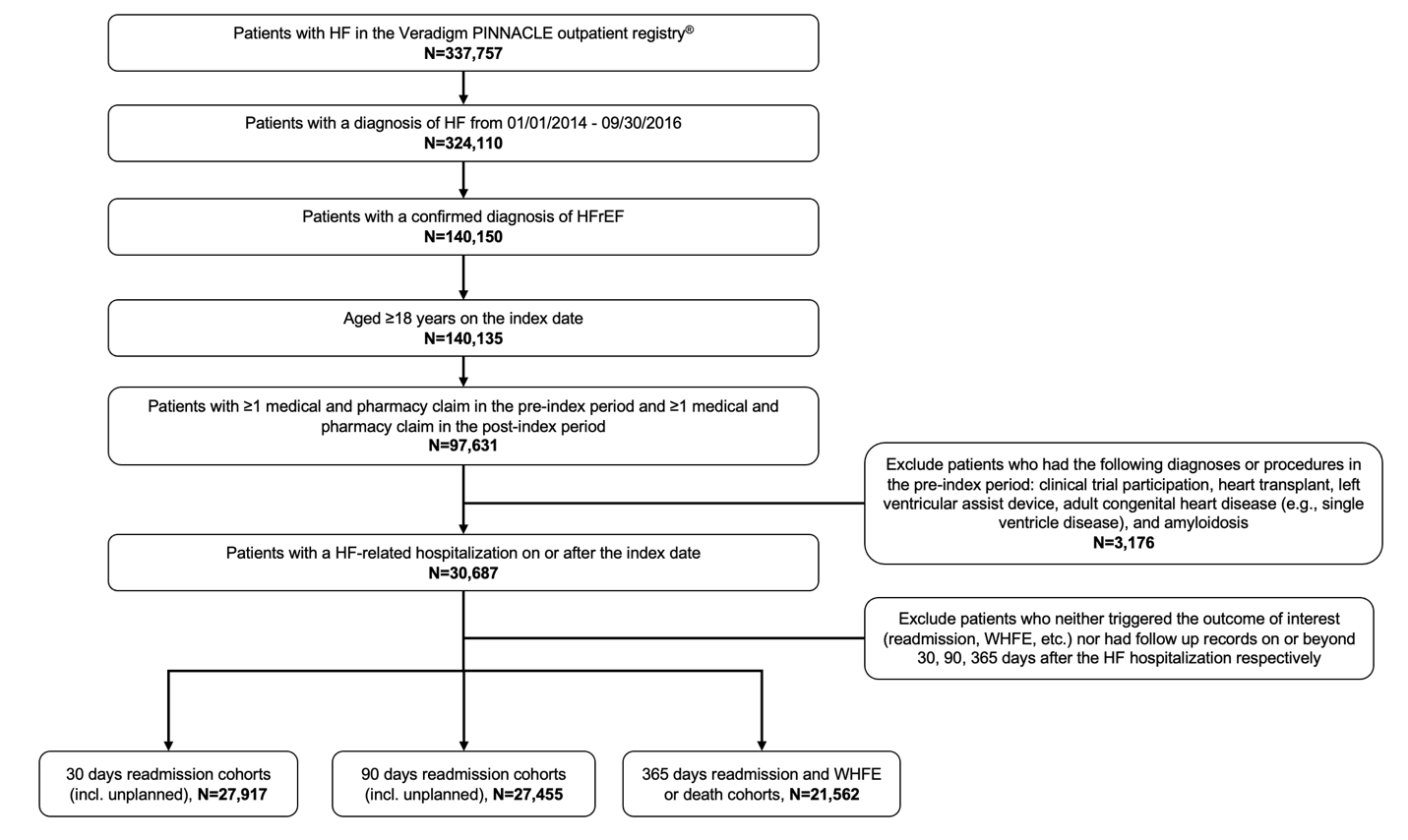


HF, heart failure; HFrEF, heart failure with reduced ejection fraction; WHFE, worsening heart failure event
