# Supplementary material for: Comparison of Machine Learning Algorithms for Predicting Hospital Readmissions and Worsening Heart Failure Events in Patients With Heart Failure With Reduced Ejection Fraction: Modeling Study
Source: JMIR Form Res. 2023 Apr 17;7:e41775. doi: 10.2196/41775 (PMC10152335; doi:10.2196/41775)
Supplement: Multimedia Appendix 2 [file formative_v7i1e41775_app2.docx]

**Appendix 2. Descriptive results of outcome measures**

| **Outcomes** | **n/total N (%)** |
| --- | --- |
| **30-day readmission** |  |
| All | 3,186/27,917 (11.4%) |
| Unplanned | 3,135/27,917 (11.2%) |
| **90-day readmission** |  |
| All | 5,792/27,455 (21.1%) |
| Unplanned | 5,710/27,455 (20.8%) |
| **365-day readmission** |  |
| All | 8,394/21,562 (38.9%) |
| Unplanned | 8,315/21,562 (38.5%) |
| All readmission or death | 9,038/21,562 (41.9%) |
| WHFE | 9,231/21,562 (42.8%) |
| WHFE or death | 9,585/21,562 (44.5%) |

WHFE, worsening heart failure event

Percentages may not add up to 100% due to rounding errors. Note, 2,770 were excluded from the 30-day readmission calculations due to a lack of follow-up on or beyond 30 days (thus, the denominator is 30,687-2,770= 27,917). For this same reason, 3,232 patients were excluded from the 90-day readmission outcome analyses and 9,125 patients were excluded from the 365 days readmission and WHFE analyses.
